# Atypical Femur Fracture in a Teenager on Chronic Imatinib Therapy

**DOI:** 10.1155/crom/2081905

**Published:** 2025-01-06

**Authors:** Ana C. Belzarena, James L. Cook

**Affiliations:** ^1^Missouri Orthopaedic Institute, Department of Orthopaedic Surgery, University of Missouri, Columbia, Missouri, USA; ^2^Orthopaedic Surgery Department, University of Missouri, Columbia, Missouri, USA

**Keywords:** atypical fracture, chronic myelogenous leukemia, imatinib, pathological fracture

## Abstract

Atypical femoral fractures (AFFs) are rare fractures usually associated with medications that can ultimately alter bone metabolism. Imatinib, a drug prescribed for treatment of chronic myeloid leukemia (CML), has been associated with altered bone homeostasis, however, with unknown clinical significance. Here, we present the case of a 17-year-old female, with a diagnosis of CML undergoing chronic imatinib therapy, who developed an AFF treated successfully with prophylactic fixation with intramedullary nailing. Our case underscores the importance of prompt recognition of this entity to allow patients timely appropriate care, even at an early age.

## 1. Introduction

Chronic myelogenous leukemia (CML) is a hematologic malignancy caused by a translocation between Chromosomes 9 and 22, also known as the Philadelphia (Ph) chromosome [1, 2]. This disease, more prevalent in the adult population, represents 2% of the leukemias of childhood [3]. CML had a high mortality rate until the appearance of tyrosine kinase inhibitors, a type of drug that competes for ATP-binding receptors and inhibits cell proliferation [4].

Imatinib, a drug within the tyrosine kinase inhibitors class, is now considered the first line of treatment for CML [5]. Adult patients in treatment with imatinib can eventually discontinue the drug if certain clinical and laboratorial criteria are met [6]. However, in the younger population, only a small percentage of patients meet those criteria, and even in those who do, 50% will need to resume the therapy [7]. Therefore, many patients will need to continue imatinib indefinitely [8].

Imatinib is known to alter bone metabolism and inhibit bone remodeling [9]; however, the severity of its clinical impact is unknown [10]. Imatinib has been linked to hypophosphatemia and secondary hyperparathyroidism, as means to disturb bone turnover. Nonetheless, patients undergoing imatinib therapy can have normal phosphate and parathyroid hormone levels and still present altered osseous metabolism with inhibited bone remodeling due to other unknown mechanisms [10].

Atypical femoral fractures (AFFs) are a subgroup of fractures that occur in the setting of minimal energy trauma and are usually located in the subtrochanteric region [11]. These atypical fractures are related to inhibited bone remodeling, for example, in the setting of chronic use of bisphosphonate class drugs, denosumab, or steroids [12]. Cases of AFFs in teenagers are extremely rare with scarce case reports in the literature [13]. Furthermore, the current literature contains sparse case reports on AFFs due to imatinib therapy, and those are described on sexagenarian patients [14].

Here, we describe the case of a 17-year-old female, undergoing chronic imatinib therapy due to CML, who developed an AFF treated successfully with prophylactic fixation with intramedullary nailing.

## 2. Case Report

**Figure 1:**
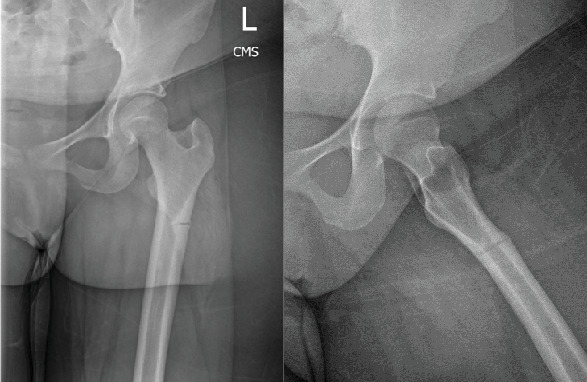
Anteroposterior and lateral radiograph of the left femur demonstrating an acute versus subacute incomplete transverse fracture of the subtrochanteric zone.

A 17-year-old Caucasian female was seen in our clinic due to left proximal femur pain. The patient stated she had chronic intermittent pain of moderate intensity for the past year, which worsened in the last 3–4 months. Recently, she had a low-energy fall from standing height which aggravated her pain acutely and prompted an urgent care visit. During this visit, a radiograph of her left femur was obtained, which demonstrated lateral cortical thickening and an incomplete transverse fracture line in a skeletally mature femur (Figure 1). In the same radiograph, normal angles of lateral bowing (3°) and anterior bowing (9°) were noticed. Furthermore, recent laboratory exams of the patient demonstrated normal phosphorus (3.4 mg/dL) and calcium (9.4 mg/dL) levels, decreased 25-hydroxy (OH) vitamin D (15.00 ng/mL), increased alkaline phosphatase (154 units/L), and normal parathyroid hormone (30 pg/mL). Additional laboratory values within range included ionized calcium 1.16 mmol/L, ionized magnesium 0.54 mmol/L, third-generation TSH 1.280 mcunit/mL, and free thyroxine 127 mg/dL. Other markers of bone metabolism were not requested at the time.

**Figure 2:**
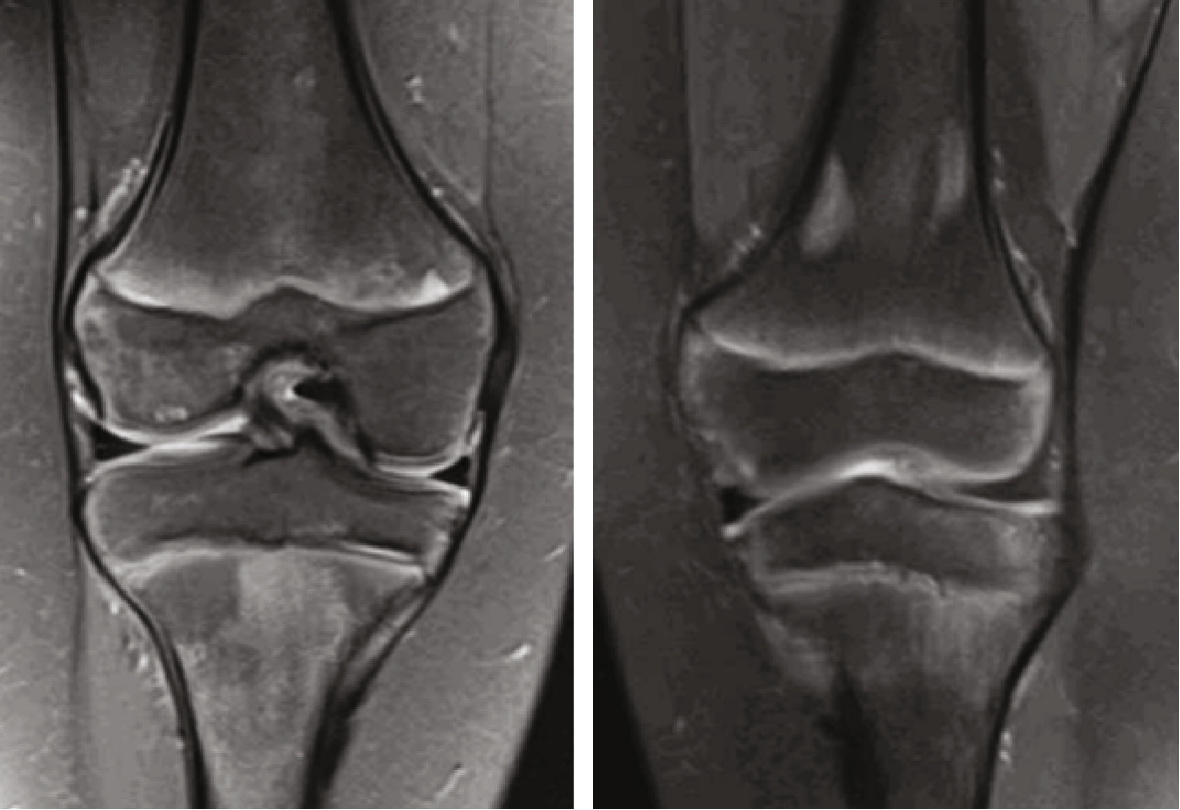
MRI of bilateral distal femora, PD TSE FS sequence, coronal view, demonstrating serpentine line of abnormal signal intensity with surrounding edema in subcortical bone of the lateral femoral condyle, consistent with avascular necrosis.

**Figure 3:**
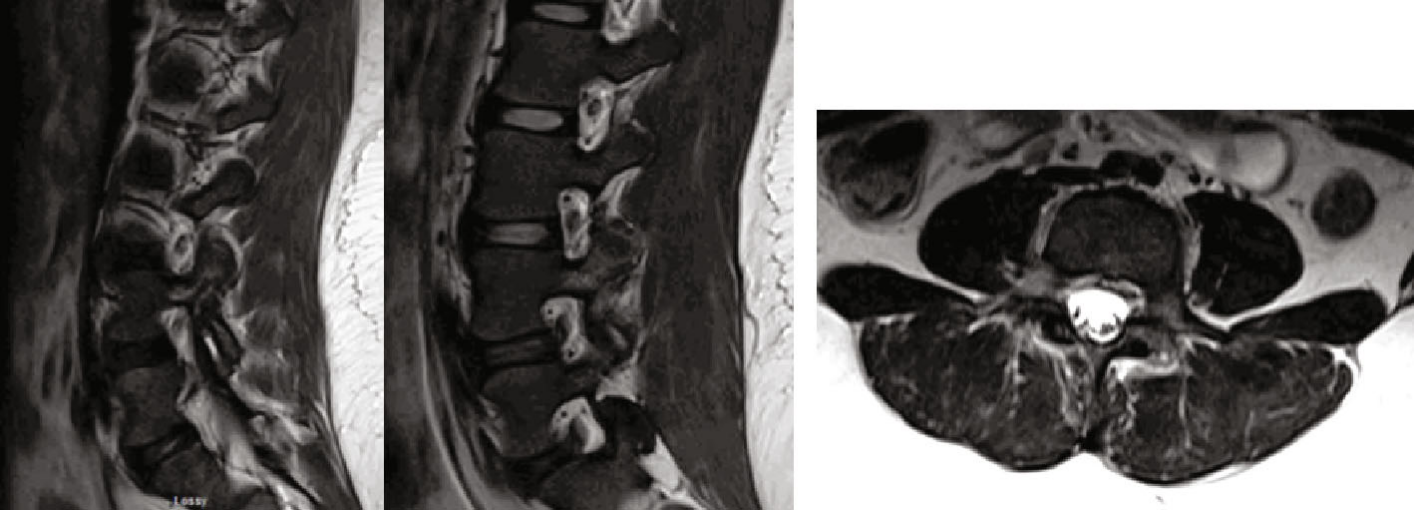
MRI without contrast, T2 sequence, sagittal and axial cut, with a bilateral L4 pars defect with associated bone marrow edema.

**Figure 4:**
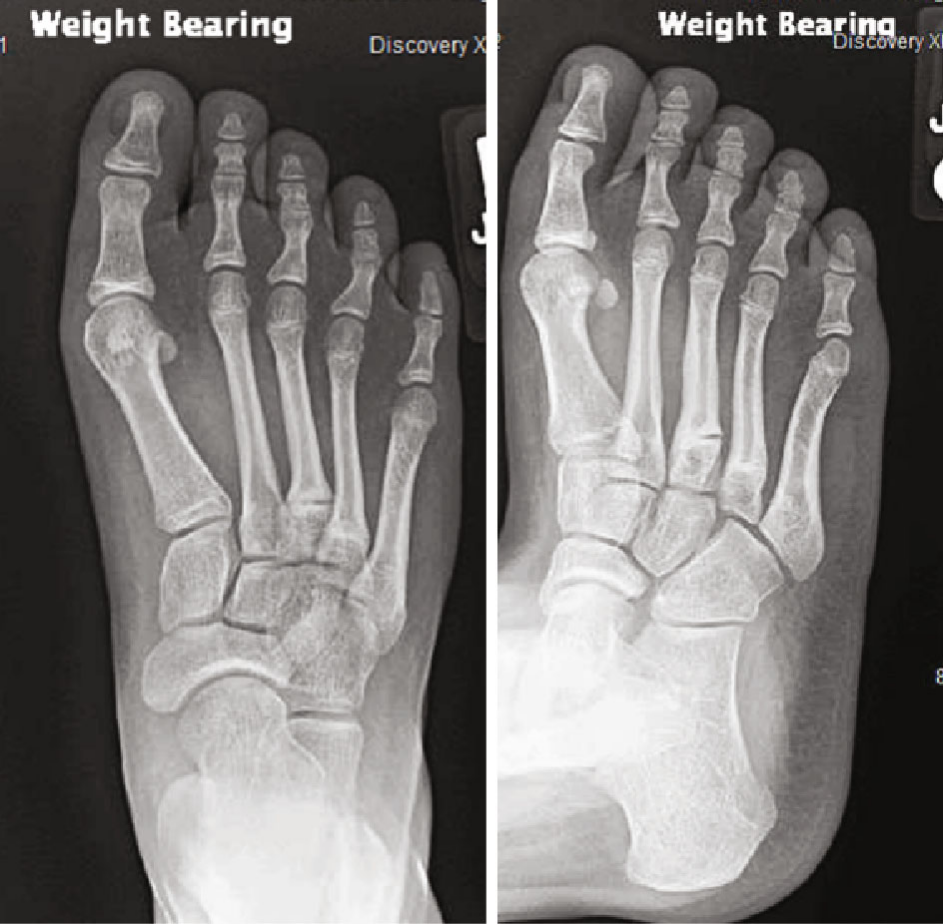
Radiographs of the right foot showing chronic incomplete fractures of the shafts of the third and fourth metatarsals.

As relevant prior medical history, the patient was diagnosed with CML at 2 years of age and was at the time of our consultation undergoing chronic imatinib therapy at a 400 mg/daily dose. Eight years after her CML diagnosis and while being on imatinib therapy, she was diagnosed with bilateral distal femur avascular necrosis (Figure 2). This was treated conservatively with pain control and physical therapy. Additionally, 12 years after diagnosis, she developed lower back pain. Imaging studies at the time demonstrated L4 bilateral pars defects with bone marrow edema (Figure 3). This was also treated conservatively with clinical improvement. In the same year, the patient developed atraumatic foot pain, which prompted more radiographs. She was then diagnosed with fractures of the third and fourth metatarsal shafts (Figure 4). She was treated with a cam boot and improved clinically; however, the fractures remained radiographically not healed.

**Figure 5:**
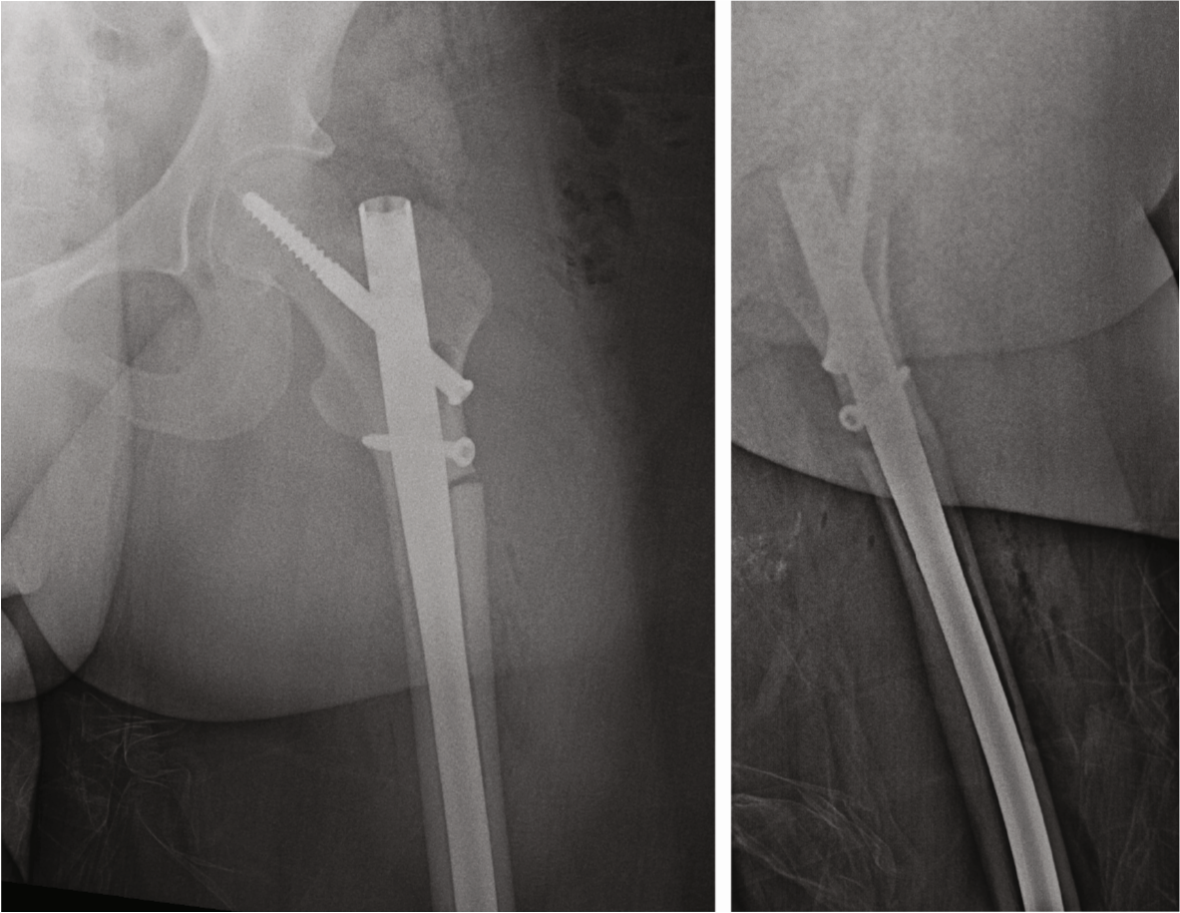
Anteroposterior and lateral radiograph of the left femur demonstrating internal fixation of the subtrochanteric atypical fracture.

**Figure 6:**
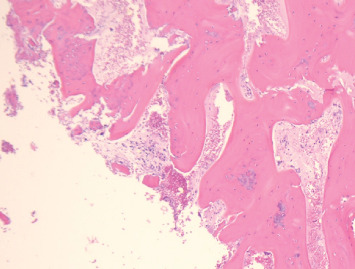
Microscopic image H&E × 10 demonstrating bone trabeculae and bone marrow without conspicuous abnormalities and no evidence of atypical cells. Limited presence of fibrosis interspersed among the bone trabeculae, suggesting that a reparative process or chronicity was also noted in the sample.

**Figure 7:**
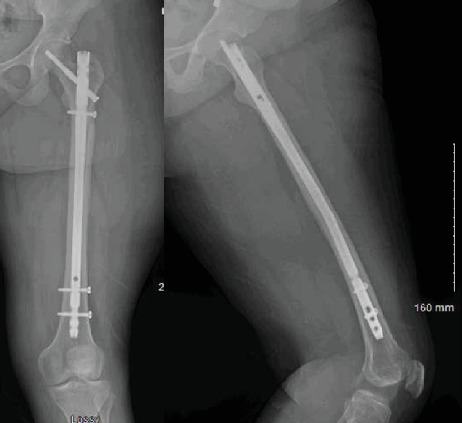
Anteroposterior and lateral radiograph of the left femur demonstrating healing of the previously noted fracture.

At the time of the patient's consultation with our service, she was examined and noted to be limping and having moderate pain and tenderness over her left proximal femur. She was also unable to do a straight leg raise with the injured leg. We explained the radiographic findings and the need to act promptly and prophylactically to avoid a complete AFF. The patient and family consented, and we proceeded with fixation with intramedullary nailing. We performed a trochanteric entry long cephalomedullary femoral nail manufactured by DePuy Synthes (West Chester, Pennsylvania, United States) (Figure 5). Samples of the femur obtained during the surgery were sent for pathology analysis and demonstrated viable cortical and trabecular bone architecture, with preserved osteocyte lacunae and no evidence of necrosis. The bone marrow displayed normal fat distribution, characteristic of hematopoietic marrow, without signs of adipocyte hypertrophy or replacement. There was presence of fibrosis interspersed among the bone trabeculae, suggesting a reparative process or chronicity. The findings are consistent with a healing response in the context of an AFF, lacking signs of acute inflammation or malignancy (Figure 6). The postoperative protocol included continuing weight-bearing as tolerated with crutches for the first 2 weeks. Physical therapy was started at that point, and the crutches slowly weaned off. At the 4-month follow-up from surgery, the patient was pain-free and walking without any assistive device. At the 9-month follow-up, the radiographs demonstrated evident signs of fracture healing with an imperceptible fracture line (Figure 7).

## 3. Discussion

Imatinib has changed dramatically the outcome of patients with CML; however, this drug is not without side effects [15]. The case we present has the uniqueness of referring to a rare orthopedic complication, consequence of the side effects of imatinib, in the setting of a young patient with CML that cannot discontinue the treatment. Furthermore, AFFs can be difficult to diagnose and challenging to treat, which emphasizes the relevance of prompt referral to an oncology orthopedics service for these exceptional cases [16].

CML, a malignancy of the blood cell lineage, has an incidence of 150 new cases a year in children in the United States and presents with fatigue, weakness, repetitive infections, and easy bleeding [17, 18]. Previous literature has reported on the side effects of imatinib, the first-line therapy for CML, on bone metabolism [9, 19]. The mechanism that leads to this alteration is not fully understood nor are the long-term osseous effects of the drug [10, 20, 21]. One of the hypotheses presented is that imatinib induces phosphorus excretion, which leads to hypophosphatemia and secondary hypoparathyroidism, proven causes of altered bone homeostasis [22, 23]. This highlights the importance of assessing patients' laboratory levels of bone metabolism markers. Nonetheless, bone alterations can still occur in the setting of normal laboratory values such as the case of our patient that had normal phosphate and PTH [10].

AFFs tend to occur in a select patient population comprised of adult patients undergoing chronic osseous antiresorptive drug therapy, for example, with bisphosphonates; these drugs are indicated for osteoporosis or cancer that has spread to bones [24, 25]. To the best of our knowledge, there is only one case in the literature describing the association of imatinib and AFFs in a 60-year-old patient [14].

Pain presents as an alarming sign in 70% of AFF patients [11]. As in our patient, she had prior intermittent pain that was initially misinterpreted as muscle pain, as well as a series of previous pathological fractures that were an omen to her AFF. Important radiographic findings of AFFs include transverse fracture line initiating on the lateral cortex, thickened cortices, cortical breaking, and periosteal thickening; however, those can be mild and easily overlooked when not assessed by a specialist [26, 27].

The natural history of these fractures, including diminished callus and bone repair, makes early diagnosis and treatment critical [28]. Completeness of the fracture line as well as presence of pain, indicates the need for surgical fixation. Nonoperative treatment oftentimes can render unsuccessful outcomes, and prophylactic fixation has an important role for these patients [16, 29]. Additionally, when a patient undergoing bisphosphonate therapy is diagnosed with an AFF, the bisphosphonates are discontinued [11, 30]. However, in the case of our patient, imatinib treatment cannot be interrupted to maintain control of the CML [8]. This makes this case even more challenging and exigent, possibly requiring continuous surveillance by a specialized team.

## 4. Conclusion

In conclusion, the occurrence of AFFs linked to chronic imatinib therapy transcends age, affecting even young patients. Vigilance towards patient symptoms, coupled with awareness of classic radiographic findings, empowers multidisciplinary teams to swiftly identify and manage these complex fractures. Such proactive approaches are paramount in ensuring optimal patient outcomes and mitigating the impact of these challenging fractures, especially in patients with CML, where imatinib therapy cannot be discontinued.

## Data Availability

The data that support the findings of this study are available on request from the corresponding author. The data are not publicly available due to privacy or ethical restrictions.
